# Novel Stemness-Related Gene Signature Predicting Prognosis and Indicating a Different Immune Microenvironment in HNSCC

**DOI:** 10.3389/fgene.2022.822115

**Published:** 2022-03-14

**Authors:** Yi Luo, Wei-Bo Xu, Ben Ma, Yu Wang

**Affiliations:** ^1^ Department of Oncology, Shanghai Medical College, Fudan University, Shanghai, China; ^2^ Department of Head and Neck Surgery, Shanghai Cancer Center, Fudan University, Shanghai, China

**Keywords:** head and neck squamous cell carcinomas, stemness, prediction, immune cell infiltration, bioinformatics, TCGA

## Abstract

**Background:** The head and neck squamous cell carcinomas (HNSCC) is one of the most frequent cancers in the world, with an unfavorable prognosis. Cancer stem cells (CSCs) have been found to be responsible for HNSCC recurrence and therapeutic resistance.

**Methods:** The stemness of HNSCC was measured using a stemness index based on mRNA expression (mRNAsi). Stemness-related genes were discovered using weighted gene co-expression network analysis, least absolute shrinkage and selection operator analysis, and Cox regression, and a stemness-related prognostic index (SPI) was constructed. This research was based on TCGA and GSE65858.

**Results:** Stemness was found upregulated in HNSCC compared with normal tissues. The risk score model including five stemness-related genes exhibited a good accuracy in predicting outcomes. High SPI predicted a shorter overall survival (OS) in HNSCC patients, in the meantime, also demonstrated a lower CD8^+^ T cell infiltration and a higher enrichment of macrophages and fibroblasts than the low-SPI group, focusing on several up-regulated pathways such as epithelial mesenchymal transition (EMT), MYC targets v1, E2F targets, mTORC1 signaling, hypoxia, MYC targets v2, angiogenesis, G2M checkpoint, and glycolysis.

**Conclusion:** The SPI signature, which includes five stemness-related genes, could be utilized as a prognostic biomarker for HNSCC, implying that stemness may impact HNSCC immunologic profiles and be a feasible therapeutic target.

## Introduction

Head and neck squamous cell carcinoma (HNSCC), one of the most common cancers worldwide, include a wide range of phenotypes such as cancers of the tongue, oral cavity, nasopharynx, oropharynx, larynx, and hypopharynx. HNSCC patients generally present with a locally advanced stage, and a significant proportion of them undergo surgery as well as combined treatment such as chemotherapy, radiotherapy, and molecular targeted therapies if metastatic and/or recurrent disease is present. HNSCC is said to have a poor prognosis, with a 5-year survival rate of 40–50%, and patients with advanced disease only having a 34.9 percent survival rate (Leemans et al., 2011).

Cancer stem cell research has revealed the unique function of cancer stem cells, which are defined as cells with stem cell characteristics, the capacity for self-renewal, and the ability to promote tumor cells to invade and grow in cancer (Korkaya and Wicha, 2010). They have the ability to produce all cell types. Furthermore, because of their distinct characteristics, cancer stem cells appear to be less susceptible to chemotherapy. Many studies have also found that cancer stem cells play an important role in cancer metastasis and differentiation (Friedmann Morvinski and Verma, 2014; Shibue and Weinberg, 2017). Cancer stem cells were found in HNSCC bulk tumors and gave rise to new tumors in immunodeficient mice, which may shed light on how residual stem cells cause tumor recurrence and regrowth in patients after treatment (Prince et al., 2007; Okamoto et al., 2009). Thus, to improve the therapeutic efficacy of HNSCC, a thorough understanding of cancer stem cells is required.

Malta et al. developed a scoring system using the one-class logistic regression (OCLR) machine learning algorithm to compare the similarity between tumor cells and different types of stem cells obtained from the Progenitor Cell Biology Consortium (https://www.synapse.org/pcbc) in order to better understand and describe the unique characteristics of cancer stem cells, and thus obtained two stemness indexes, mDNAsi and mRNAsi (Malta et al., 2018), which were estimated based on the level of DNA methylation and mRNA expression, respectively. The importance of stemness-related indicators in solid tumors is becoming recognized, while their involvement in the risk of HNSCC has yet to be determined.

In this work, we conducted an integrative analysis to create a five-gene signature for predicting prognosis in patients with squamous cell carcinoma of the head and neck (HNSCC). In the TCGA-HNSC datasets, WGCNA, Cox regression, and least absolute shrinkage and selection operator analysis (LASSO) regression analysis revealed five genes (SPOCK1, BOC, KNSTRN, MME, and GRIA3). Based on the stemness-score-related prognostic index (SPI), the immune landscape was visualized and GSEA revealed the associated functional signaling pathways.

## Materials and Methods

### Data Preparation

The RNA-Seq data of 499 HNSCC patients was obtained from The Cancer Genome Atlas (TCGA) database (https://cancergenome.nih.gov/), together with the related clinical information such as age, gender, American Joint Committee on Cancer (AJCC) stage, and survival statistics, meanwhile, gene expression profiles and survival data from 270 GSE65858 HNSCC patients were acquired from the Gene Expression Omnibus (GEO) database (http://www.ncbi.nlm.nih.gov/geo/) (Supplementary Table S1). Each gene’s expression level was log2 transformed, and the average value was calculated for many probes corresponding to the same gene. Paired differential gene analysis was done in 43 HNSCC patients from the TCGA, with a false discovery rate (FDR) of 0.05 and an absolute of log2 fold change > 1 utilized for filtering of differentially expressed genes (DEGs). Meanwhile, the Progenitor Cell Biology Consortium (PCBC) database expression data of pluripotent stem cells (embryonic stem cells and induced pluripotent stem cells) were evaluated, and the one-class logistic regression (OCLR) method was used to predict mRNA expression-based stemness score.

### Weighted Gene Co-Expression Network Analysis

WGCNA is a valuable approach for identifying modules of highly correlated genes and investigating the substantial relationship between modules and external sample features. A scale-free co-expression network was built in the study to investigate stemness-score-related genes. As a soft-threshold parameter, a power of = 7 with a scale-free R2 > 0.90 was chosen. The expression matrix was transformed into an adjacency matrix, which was then transformed into a topological overlap matrix (TOM). To cluster genes based on TOM, average linkage hierarchical clustering was performed. The gene dendrogram’s minimum genome number was 50, and nongray modules were detected by setting the merging threshold function to 0.25. The module eigengenes (MEs) were defined as the primary component of each module’s gene expression matrix. The association between MEs and stemness score revealed the relevant module. As a consequence, one highly correlated module (brown) and two highly correlated modules (yellow and green) were discovered.

### Prognostic Gene Screening and Validation

To begin, univariate cox regression and Pearson correlation analysis were utilized to identify the statistically significant stemness-related prognostic genes, using all genes in the brown, yellow, and green modules to show the strong link to prognosis in TCGA HNSCC. The 499 TCGA tumor samples were then randomly split into training and validation cohorts in a 2:1 ratio. The LASSO regression with 10-fold cross-validation was then used to filter prognostic genes, and a stemness-related prognostic index (SPI) was constructed using regression coefficients from the multivariate cox regression in the trial set, where the Akaike information criterion (AIC) was utilized to optimize the data. Finally, five genes were found and the formula of the risk score model was described as:

SPI=∑i Coefficient (mRNAi)×Expression (mRNAi) 

Following that, the model was validated using the TCGA inner validation set and the GSE65858 outer validation set from the GEO dataset. In addition, clinical parameters such as age, gender, and TNM stage were included in the multivariate cox regression model to explore the importance of SPI. The signature’s prognostic efficiency was evaluated using a time-dependent receiver operating characteristic (ROC) curve.

### Clinical Correlation

The Pearson’s correlation was used to depict the association between stemness score and five genes. The Kaplan–Meier curve was used for each stemness score-related gene signature in the TCGA cohort, and it clearly distinguished between high and low expression groups. Following that, the relationship between various clinical features and SPI was investigated.

### Detection of Gene Expression

The proteins encoded by the five stemness-related genes were studied using clinical samples from the Human Protein Atlas (HPA) database (https://www.proteinatlas.org).

### Analysis of Immune Status in SPI Groups

The CIBERSORT (Newman et al., 2015), CIBERSORT-ABS (Newman et al., 2015), QUANTISEQ (Finotello et al., 2019), XCELL (Aran et al., 2017), MCP-counter (Becht et al., 2016), EPIC (Racle et al., 2017), and TIMER2.0 (Li et al., 2020) algorithms were used to compare the proportion of tumor-infiltrating immune cells between the high and low SPI risk groups based on the five gene signatures discovered.

### Gene Enrichment Analysis

In the TCGA cohort, “fgsea” was used for the GSEA analysis to compare the KEGG functional enrichment analysis in the high-SPI group to the low-risk group.

### Statistical Analyses

R was used to conduct all statistical analyses (Version 4.1.0). The Kaplan-Meier method and the log-rank test were applied to evaluate the prognostic impact of core genes and signature score on the overall survival (OS). Statistical significance of continuous parameters was assessed using either the student’s *t* test or the Wilcox test, depending on whether the data was regularly distributed or not. A *p* value of 0.05 was deemed statistically significant.

## Results

### Co-Expression Network Construction

**FIGURE 1:**
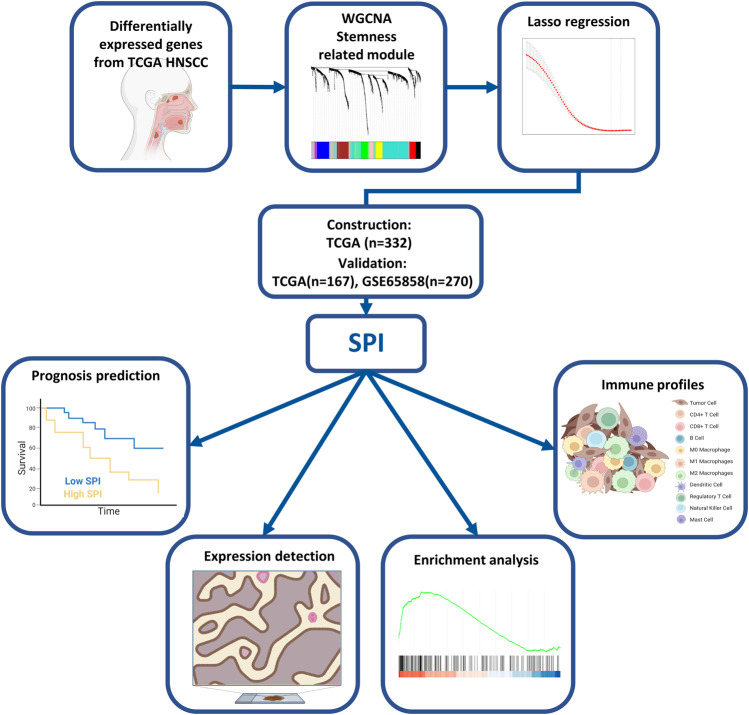
Flowchart of the study.

**FIGURE 2:**
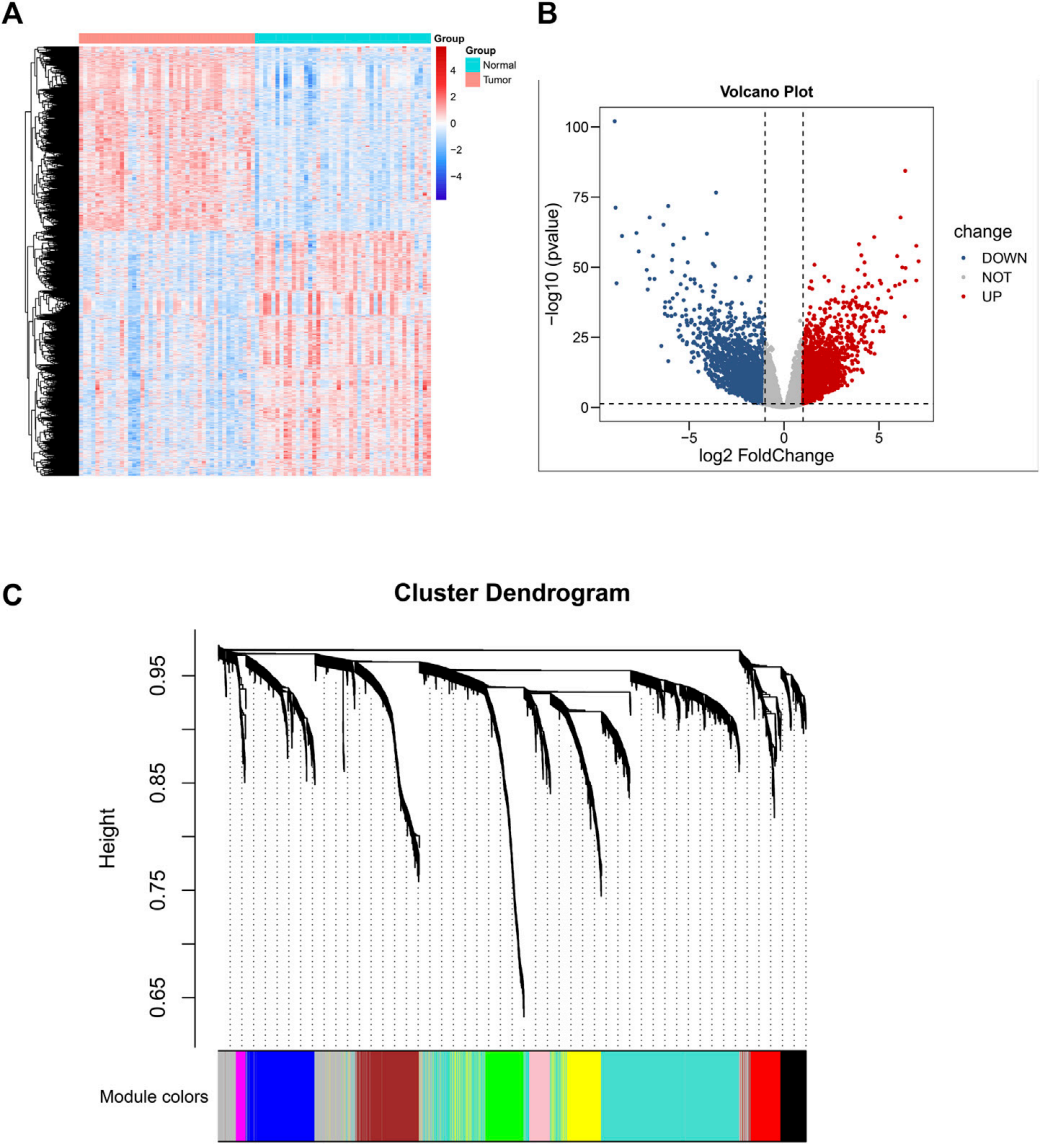
Differential gene expression analysis based on 43 normal-tissue-matched HNSCC patients and construction of co-expression network. **(A)** Heatmap of differentiated expressed genes (DEGS). **(B)** Volcano plot of DEGS. **(C)** A cluster diagram of gene cluster of HNSCC.

The research constructure was shown in Figure 1. PCBC was used to obtain 78 examples of expression data from pluripotent stem cells. After that, it was discovered that the mRNA expression-based stemness score in TCGA HNSCC tissues was substantially greater than that of normal tissues (*p* < 0.05) (Supplementary Figure S1), which, however, had no statistical significance in OS (*p* = 0.84) (Supplementary Figure S2). Therefore, we conducted an integrated analysis to find a more valuable gene signature based on stemness score. First, according to TCGA data, differential gene analysis was done in 43 tumor and matched normal tissues to look for significantly different expressed genes. In all, 4,197 DEGs were discovered, comprising 2,391 down-regulated genes and 1806 up-regulated genes (Figures 2A,B). Then, the resulting expression matrix of HNSCC samples in TCGA data was then utilized to perform WGCNA analysis. To quantify the distance between each gene, a Pearson correlation matrix was constructed. We chose = 7 as the soft-threshold for constructing a matrix of similarity among all pairs of genes (Supplementary Figure S3). Then, applying average linkage hierarchical clustering, a variety of gene modules were selected out (Figure 2C).

### Significant Module Identification

**FIGURE 3:**
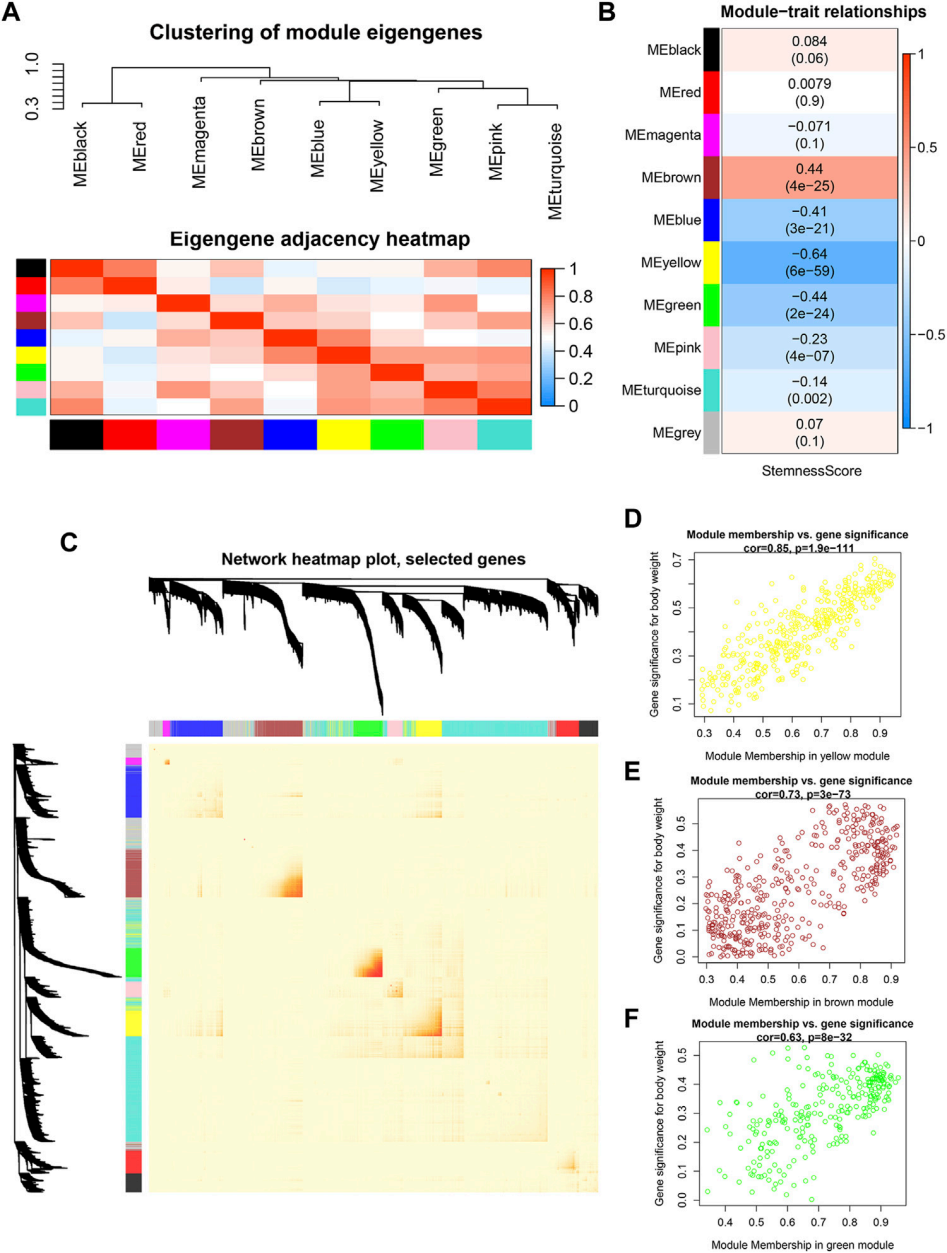
Identification of modules significantly correlated to stemness **(A)** Heatmap of the adjacencies in the hub gene network. **(B)** Heatmap of the correlation between module eigengenes and the stemness score. **(C)** Interaction relationship of co-expression genes. **(D–F)** Scatterplot of module eigengenes in yellow, brown and green modules.

To investigate co-expression similarity, we computed and grouped eigengenes based on their correlations, and the results were displayed (Figure 3A). We next used statistical analysis to discover modules that are closely associated to stemness score in order to uncover genes with stemness associations. The module eigengene of the yellow module was shown to have the strongest linkage with stemness score in HNSCC patients, followed by the brown module and green module (Figure 3B). The interactive relationships of the gene modules were computed and clustered in a heatmap, and it was discovered that brown, green, and yellow modules exhibited strong co-expression gene interaction interactions (Figure 3C). Figures 3D–F showed the associations between module membership and gene importance in the yellow, brown, and green modules, respectively.

### Identification of Stemness-Related Genes and Construction of a Prognostic Model

**FIGURE 4:**
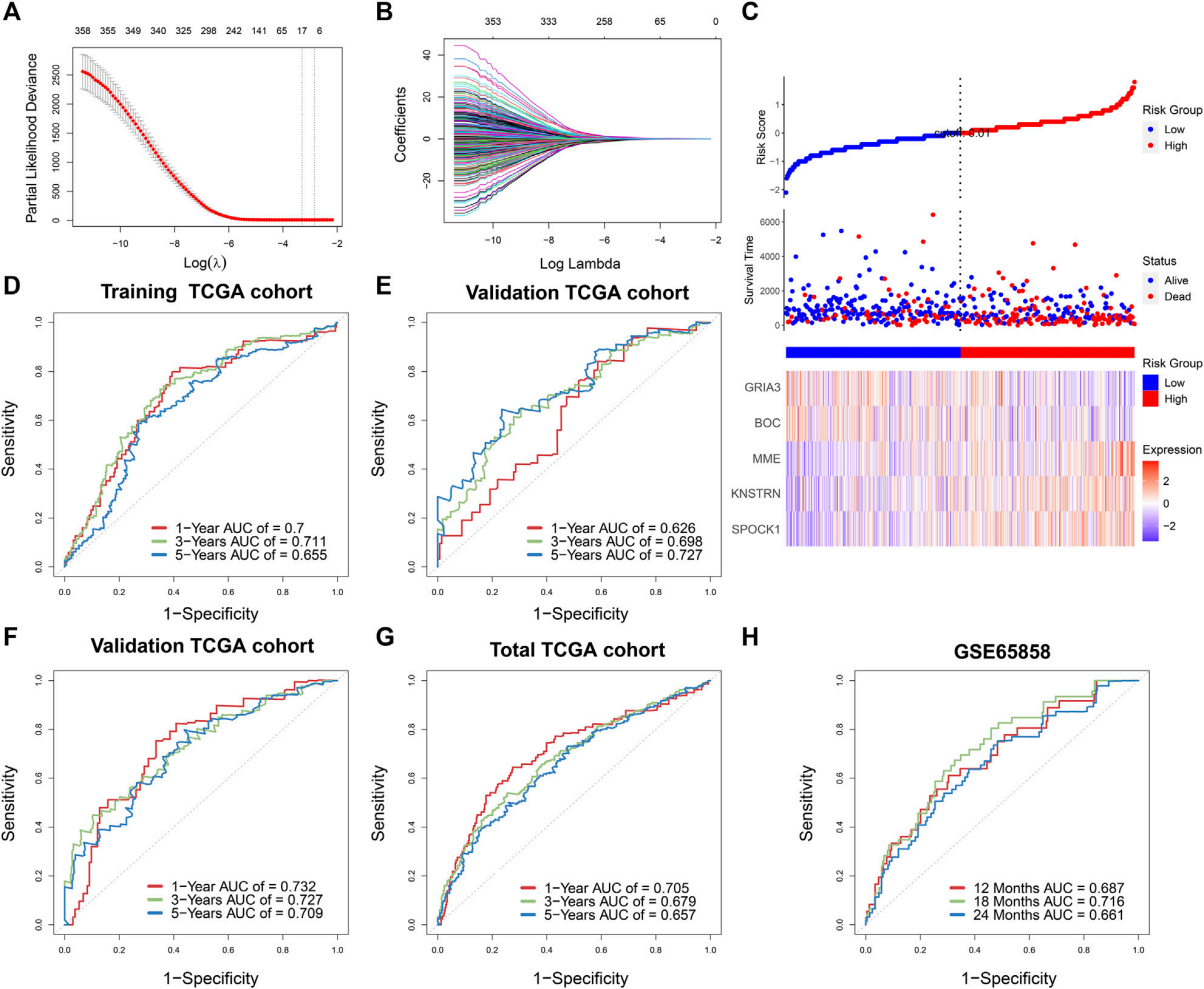
**(A)** Stemness-related genes were selected by the least absolute shrinkage and selection operator (LASSO) regression model according to minimum criteria. **(B)** The coefficient of stemness-related genes was calculated by LASSO regression. **(C)** Risk score distribution in 499 TCGA-HNSCC based on the five stemness-related genes. Area under time-dependent receiver operating characteristic curve (AUC) verified the prognostic accuracy of stemness prognostic index (SPI) in the training (*n* = 332) **(D)** and validating (*n* = 167) TCGA cohort **(E)**. AUC verified the prognostic accuracy of multivariate Cox regression module including SPI, age, gender, T stage, N stage, and M stage in the validating TCGA cohort (*n* = 167) **(F)**, the total TCGA cohort (*n* = 499) **(G)**, and the external GSE65858 cohort (*n* = 270) **(H)**.

First, univariable Cox regression was used to filter prognostic genes from significant WGCNA modules, which included 395 genes in the yellow module, 433 genes in the brown module, and 275 genes in the green module. Second, 226 potential prognostic genes were identified, and correlation analysis was performed to further identify the significant stemness-related genes statistically (|Pearson correlation coefficient| > 0.4, *p* < 0.05). Third, LASSO regression was done using 10-fold cross-validation to further filter the results (Figures 4A,B). Finally, the identification of five genes signature (SPOCK1, BOC, KNSTRN, MME, and GRIA3) was assisted by the lowest AIC score in the multivariate Cox regression analysis. The SPI was calculated using the expression levels of each core gene as well as the coefficient from the multivariate Cox regression.

SPI=0.17346×SPOCK1−0.29808×BOC+0.26439×KNSTRN+0.18580×MME−0.15037×GRIA3  

The survival result, risk status, and core gene expression levels of 499 HNSCC patients from the TCGA were presented, and patients in the high-SPI group had a larger risk of mortality than patients in the low-SPI group (Figure 4C). The area under ROC curve (AUC) of the training set was 0.700 at 1 year, 0.711 at 3 years and 0.655 at 5 years (Figure 4D). The results of multivariate Cox regression of five genes signature were also shown (Supplementary Table S2).

### Validation of the Prognostic Model

According to the formula derived in the training cohort, we estimated the SPI of the TCGA internal validation cohort (*n* = 167) and an outside validation cohort of GSE65858 (*n* = 270). Similarly, as shown in Figure 4E, the AUC of the TCGA internal validation was 0.626 at 1 year, 0.698 at 3 years and 0.727 at 5 years, respectively. Furthermore, multivariate survival analysis was performed in training and validation sets, taking into account clinical characteristics such as age, gender, and TNM stage, suggesting a substantial influence of SPI on the OS of HNSCC patients (training set: HR = 2.63, 95% CI = 1.74–3.98, *p* < 0.001; validation set of TCGA: HR = 2.14, 95% CI = 1.43–3.20, *p* < 0.001; validation set of GSE65858, HR = 1.85, 95% CI = 1.16–2.95, *p* = 0.001) (Supplementary Table S3). According to clinical variables and SPI, the AUCs of the TCGA internal validation and total TCGA cohort were 0.732 and 0.705 at 1 year, 0.727 and 0.679 at 3 years, and 0.709 and 0.657 at 5 years, respectively, (Figures 4F,G). The external validation set of GSE65858 also showed good prognostic prediction of SPI with AUCs of 0.687, 0.716, and 0.661 at 12, 18, and 24 months (Figure 4H).

### Protein Level Expression and Correlation of Stemness-Related Genes

**FIGURE 5:**
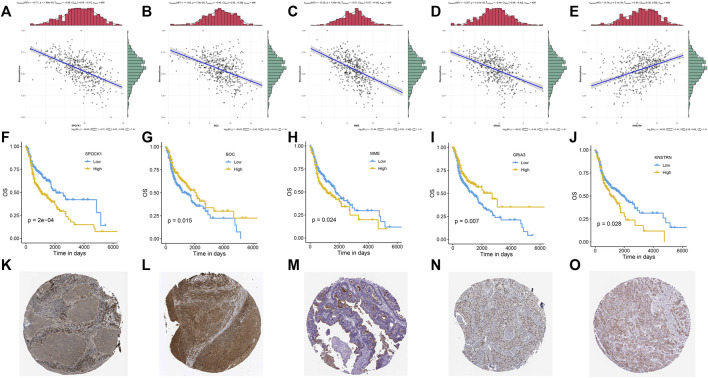
Correlations between mRNA expression and stemness score of five stemness-related genes: SPOCK1 **(A)**, BOC **(B)**, MME **(C)**, GRIA3 **(D)**, and KNSTRN **(E)**. Kaplan-Meier curves of five gene signatures: SPOCK1 **(F)**, BOC **(G)**, MME **(H)**, GRIA3 **(I)**, and KNSTRN **(J)**. The expression profiles of the proteins encoded by SPOCK1 **(K)**, BOC **(L)**, MME **(M)**, GRIA3 **(N)**, and KNSTRN **(O)**. *p* value < 0.05 was controlled.

**FIGURE 6:**
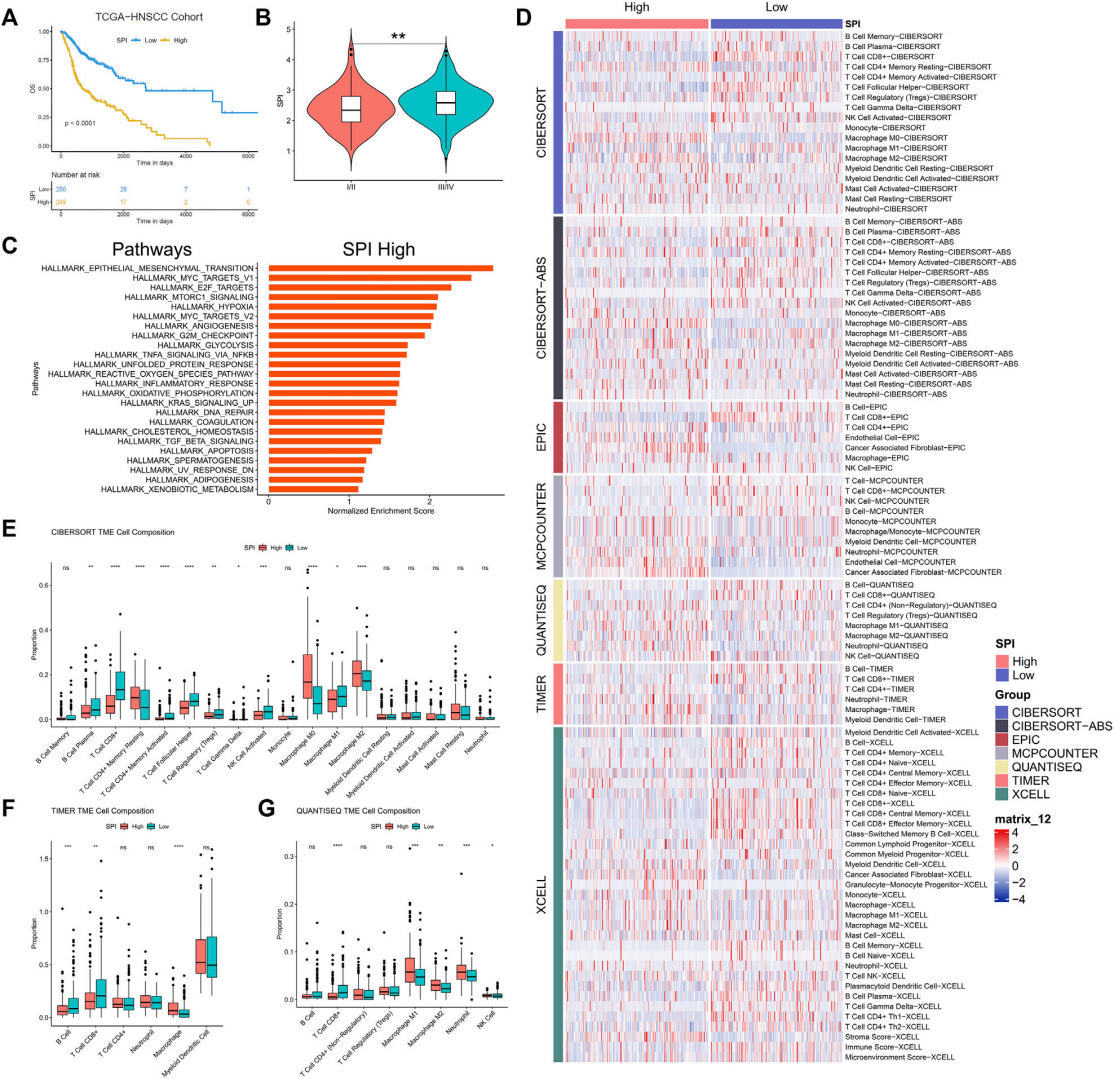
**(A)** Kaplan-Meier curves of stemness prognostic index (SPI) in HNSCC. **(B)** Correlation between stemness prognostic index (SPI) and TNM stage of HNSCC. **(C)** GSEA for expression profiles in high SPI group over low SPI group. **(D)** Heatmap for immune cell infiltration landscape based on the CIBERSORT, CIBERSORT-ABS, QUANTISEQ, XCELL, MCP-counter, EPIC, and TIMER algorithms among high and low SPI group. Box plot showed the different proportions of tumor-infiltrating cells between different SPI groups based on CIBERSORT **(E)**, TIMER **(F)**, and QUANTISEQ **(G)**. *p* value < 0.05 was controlled.

An association study was done between the stemness score based on mRNA data and the five genes identified in the investigation progress mentioned above. It was discovered that four genes, including SPOCK1, BOC, MME, and GRIA3, had a negative association with stemness score, whereas KNSTRN had a positive correlation with stemness score (Figures 5A–E). According to the findings of the Kaplan-Meier survival analysis, all five genes showed a substantial capacity to discriminate high expression group from low expression group in HNSCC patients (Figures 5F–J). Multivariate Cox regression further indicated that these five genes had strong prognostic significance (Supplementary Table S2). Besides, using clinical specimens from the HPA database, we examined the expression of the proteins produced by the stemness-related genes. SPOCK1, BOC, MME, and GRIA3, and KNSTRN were all shown to be positive in HNSCC tissue (Figures 5K-O). Kaplan–Meier plot demonstrated that SPI considerably shorten the OS of HNSCC in TCGA set (*p* < 0.01) (Figure 6A). HNSCC patients with TNM stage III/IV exhibited considerably higher SPI than those with TNM stage I/II, (Figure 6B), whereas no significant gender difference was detected between the high and low SPI groups (Supplementary Figure S4).

### KEGG Enrichment Analysis

GSEA results revealed that some signaling pathways, such as epithelial mesenchymal transition (EMT), MYC targets v1, E2F targets, mTORC1 signaling, hypoxia, MYC targets v2, angiogenesis, G2M checkpoint, and glycolysis, were up-regulated in the high-SPI group of HNSCC (false discovery rate < 0.25) (Figure 6C).

### Altered immune profiles across the Low-SPI and High-SPI groups

CIBERSORT, CIBERSORT-ABS, QUANTISEQ, XCELL, MCP-counter, EPIC, and TIMER2.0 algorithms were used to examine the immune cell and pathway profiles in the signature-identified high and low SPI groups (Figure 6D). In the preceding algorithm, we demonstrated the findings of the CIBERSORT, TIMER2.0, and QUANTISEQ algorithms used to study the immune cell infiltration landscape. SPI groups showed obvious differences in the enrichment of non-tumor cells, with a characteristic of lower infiltration of CD8^+^ T cells and higher infiltration of tumor associated macrophages in the high-SPI group compared to the low-SPI group (Figures 6E–G, Supplementary Figure S5).

## Discussion

According to a recent study, cancer stem cells play an important role in cancer growth, progression, and therapy resistance. It suggests that the role of cancer stemness in HNSCC should be investigated further. In this study, we used a series of bioinformatic algorithms to identify the gene signature of stem cells, build a prognostic prediction risk score model, perform gene enrichment analysis, and visualize immune infiltration profiles.

The process of oncogenic dedifferentiation is a critical signature in the progression of normal tissue to precancerous lesions and cancer, as evidenced by the discovery that tumor samples were more stem-like than normal samples. Certain mutations contribute to the oncogenesis of dedifferentiated cells rather than mature cells, implying that the status of cell dedifferentiation may be an oncogenic factor. Although stemness score is critical and is linked to aggressiveness of tumor, it is not a good index of prognostic prediction in HNSCC. Thus, we aimed to find a more relevant and significant gene signature. First, using WGCNA and the stemness score, we identified candidate stemness-related genes. Next, using LASSO and Cox regression, we built a prognostic risk score model which was validated both internally and externally, indicating a strong prediction ability. The SPI was calculated based the prognostic model, including BOC, GRIA3, MME, SPOCK1, and KNSTRN, which were associated with stemness, TNM stage, prognosis, and tumor microenvironment (TME). Many prominent up-regulation pathways related to tumor progression and development were discovered in high-SPI HNSCC patients compared to the low-SPI group, including EMT signaling, MYC targets v1, E2F targets, mTORC1 signaling, hypoxia signaling, MYC targets v2, angiogenesis, G2M checkpoint, and glycolysis. These well-known pathways are intricate and interactive (González-González et al., 2021), and there is a significant difference between the high-SPI group and the low-SPI group, demonstrating the utility of SPI in identifying HNSCC patients based on functional level.

The protein expression of all five genes was discovered in HNSCC using the public HPA database. BOC and GRIA3 were found to be adversely linked with stemness score and to be protective risk factors in the prognosis of HNSCC (Figures 5B,D,G,I). KNSTRN, on the other hand, had a favorable link with stemness score but a negative relationship with prognosis (Figures 5E,J). Surprisingly, MME and SPOCK1 were found to have a negative relationship with both stemness score and survival in HNSCC (Figures 5A,C,F,H).

Membrane Metalloendopeptidase (MME) is the prototype of the membrane bound zinc-dependent endopeptidase family, and it is also overexpressed in numerous malignancies, including colorectal carcinoma, pancreatic endocrine tumors, and metastatic melanomas. MME has suppressed and promoted effects on tumor development depending on the cancer types. Some suggested that low MME expression levels are associated with poor prognosis in ovarian and prostate cancers, and that MME depletion leads to Akt activation and hence contributes to cancer clinical progression (Ffrench et al., 2017; Osman et al., 2006). Moreover, it was interestingly proposed that MME collaborated with PTEN in carcinogenesis suppression by limiting the activities of prostate stem/progenitor cells (Cheng et al., 2020), which was consistent with our outcome in HNSCC (Figure 5C). Others found opposed results. For example, high MME was significantly associated with poor OS in NSCLC (*n* = 342) (Leithner et al., 2014). MME was also found to be required for proliferation of the neuroendocrine cells that were co-repressed by MENIN and DAXX, and *in vivo* experiments proved that knockdown of MME suppressing the tumor growth (Feng et al., 2017). The clinical outcome of HNSCC is related to the complicated TME consisting of tumor cells, immune cells and stroma cells (Wondergem et al., 2020). It was reported that cancer associated fibroblasts (CAFs) up-regulated MME expression under hypoxia, while NSCLC cell lines did not show a significant MME overexpression (Leithner et al., 2014). This phenomenon was also found in our study that the SPI high group of HNSCC had an up-regulated hypoxia signaling (Figure 6C), as well as increased CAFs enrichment and stroma score (Figure 6D, Supplementary Figure S5). The TCGA data was bulk RNA data, so we proposed that CAFs or others non-tumor cells in the TME of HNSCC might up-regulated MME under hypoxia stress and reveal a poor prognosis in the end. More evidence such as single-cell transcriptomics should be investigated to prove this result in the future.

SPOCK1, a secreted matricellular protein and also called SPARC, is a critical regulator of EMT induction and could be a novel therapeutic target for cancer progression (Sun et al., 2020). Previous study had confirmed the overexpression of SPOCK1 in HNSCC clinical specimens which was negatively related to the survival. Knockdown of the SPOCK1 validated its promotional effect in HNSCC cell aggressiveness. Moreover, SPOCK1 is reported promoting the proliferation and migration in many other cancers like colon cancer, pancreatic cancer and breast cancer via NF-κB pathway or AKT/mTOR pathway (Xu et al., 2020; Cui et al., 2022; Liu et al., 2021). However, SPOCK1 had a negative effect on CSCs in gastric cancer (Ma et al., 2019). Gastric CAFs derived SPOCK1 was significantly associated with tumor differentiation and suppressing SPOCK1 expression in gastric CAFs facilitated the phenotypic alteration of gastric cancer cells towards CSC-like cells where AKT/mTOR and MEK/ERK pathways might participate (Ma et al., 2019). An increased enrichment of CAFs was observed in the high SPI group and further studies are necessary to explore the source of SPOCK1 and validate its role in HNSCC progress.

The SPI was related to stemness negatively and indicated a critical role of stemness in survival of HNSCC. However, the stemness score did not show statistically significant difference in prognosis of HNSCC. The TME did not only depend on tumor cells but also was consisted of numerous non-tumors including immune cell and stroma cells. In addition, the expression of CSC makers in human tumor tissues has been demonstrated to be associated with the amount of tumor-infiltrating immune cells, implying that CSCs have a close association with the tumor immunological microenvironment (Xu et al., 2018; Sang et al., 2020). Therefore, we further investigated the TME in HNSCC based on the SPI.

CSCs originated from HNSCC have been shown to downregulate MHC molecules, which is required for a functional T-cell response against tumor cells (Chikamatsu et al., 2011). CD44^+^ HNSCC cells exhibited CSC characteristics and down-regulated HLA-A2, HLA class II, and TAP2, as well as high levels of immune modulatory cytokines such interleukin-8 (IL-8) and granulocyte colony-stimulating factor (G-CSF), indicating antigen presentation and processing dysfunction (Chikamatsu et al., 2011). SPI was calculated from five stemness-related genes. To examine the relationship between stemness and immune cell infiltration in HNSCC, we used the CIBERSORT, CIBERSORT-ABS, QUANTISEQ, XCELL, MCP-counter, EPIC, and TIMER2.0 algorithms. As a result, we demonstrated that the high-SPI group with poor prognosis had an immunologic “cold” profile (almost no immune cells visible), which was especially characterized by increased macrophages and CAFs as well as low CD8^+^ T cells and B cells (Figure 6, Supplementary Figure S5). “Cold” HNSCCs are associated with poorer OS than immunologic “hot” (immune cells in the stroma and between cancer cells) or “excluded” (immune cells at tumor boundaries) HNSCCs (Ribbat-Idel et al., 2021). And recurrent HNSCC always have significant loss of CD8^+^ T cells and B cells vs. primary tumors (Watermann et al., 2021). Interestingly, it was recently shown that CSCs of HNSCC could avoid host immune responses in a CD8^+^ T cell-dependent way (Wang et al., 2021). In HNSCC, CSCs could highly express B7-H3 to evade immune surveillance (Wang et al., 2021). Thus, low SPI HNSCC patients who have high infiltration of CD8^+^ T cells might be benefited from immune checkpoint blocker (ICB) therapy targeting B7-H3. Meanwhile, we observed that more M0 and M2 macrophages. Previous research found that M2 macrophage infiltration was negatively related to CSC marker expression and significantly associated with high tumor PD-L1 expression in oral squamous cell carcinoma (Wang et al., 2021). The comparatively high enrichment of macrophages and less both CD8+ T cells and B cells indicated an immunosuppressed TME and poor survival in the high-SPI group of HNSCC, who might be benefited from ICBs targeting PD-L1 or macrophage in the future. And more clinical trials are needed to prove this outcome.

Some studies have reported some subgroups or gene signatures that can be used to classify the clinical features and outcome of HNSCC. For example, Keck et al. used nearest centroid to identify two biologically different HPV subtypes and three non-HPV subtypes (Keck et al., 2015). The basal (BA) HNSCC subtype (well differentiated) showed a significant enrichment for hypoxia signaling and EMT signaling, as well as a relatively reduced overall survival (Keck et al., 2015). The inflamed/mesenchymal subtype (IMS), on the other hand, had a better prognosis than the BA subtype (Keck et al., 2015). Moreover, downregulation of epithelial differentiation markers was detected in IMS subset and IMS-HPV+ subtype tumors that were non-keratinizing and poorly differentiated according to morphological evaluation (Keck et al., 2015). These findings were consistent with our findings that the SPI high group has a poorer prognosis and less stemness than the SPI low group. Furthermore, our findings that the SPI low group of HNSCC has increased CD8^+^ T cells matched into the characteristic of IMS subset indicated above. To summarize, our research revealed a set of novel gene signatures based on stemness that were well-fitting to prior outcomes and had good predictive value. However, specific mechanisms behind the phenomenon needs further investigation. The study may open the approach for improved anti-tumor immunity and the development of innovative treatment approaches for HNSCC.

## Data Availability

The original contributions presented in the study are included in the article/Supplementary Material, further inquiries can be directed to the corresponding author.
