# Supplementary material for: Novel Stemness-Related Gene Signature Predicting Prognosis and Indicating a Different Immune Microenvironment in HNSCC
Source: Front Genet. 2022 Mar 14;13:822115. doi: 10.3389/fgene.2022.822115 (PMC8963956; doi:10.3389/fgene.2022.822115)
Supplement: Supplementary file 1 [file DataSheet1.docx]

Supplementary Material

# Supplementary Figures and Tables

## Supplementary Figures


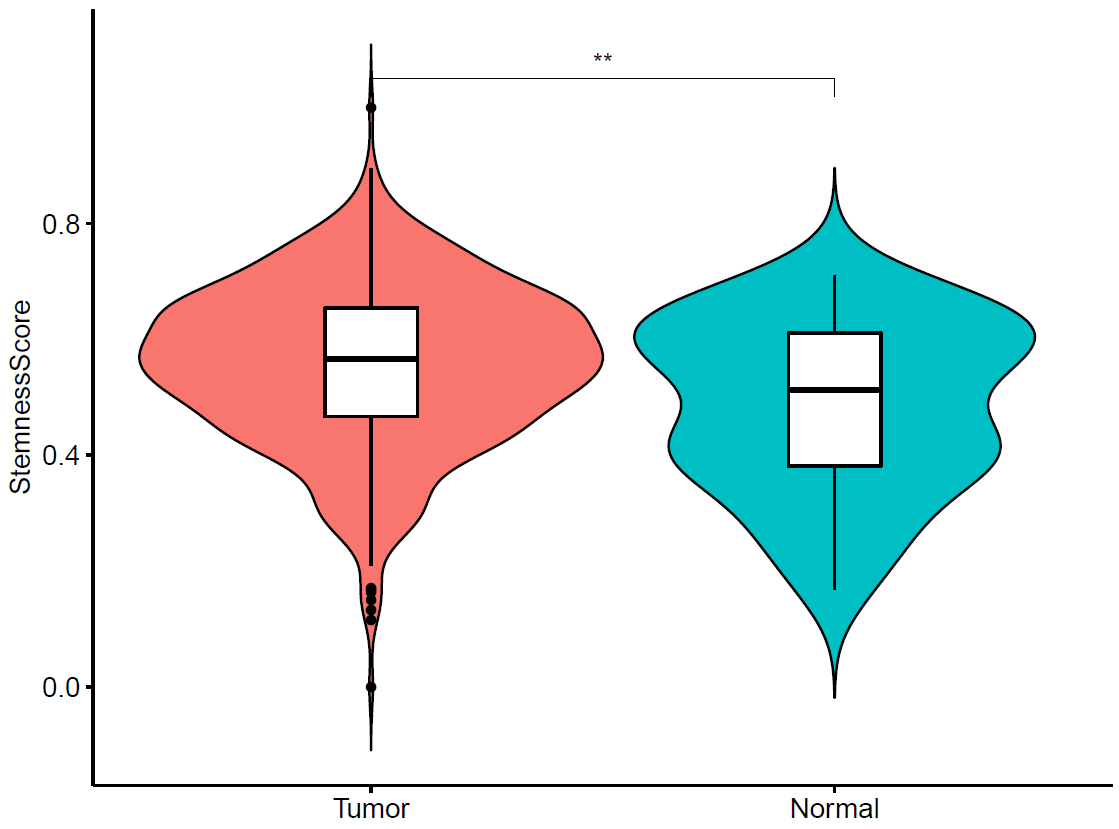


**Supplementary Figure 1.** The different expressions of stemness score based on mRNA expression between normal and tumor samples in HNSCC.


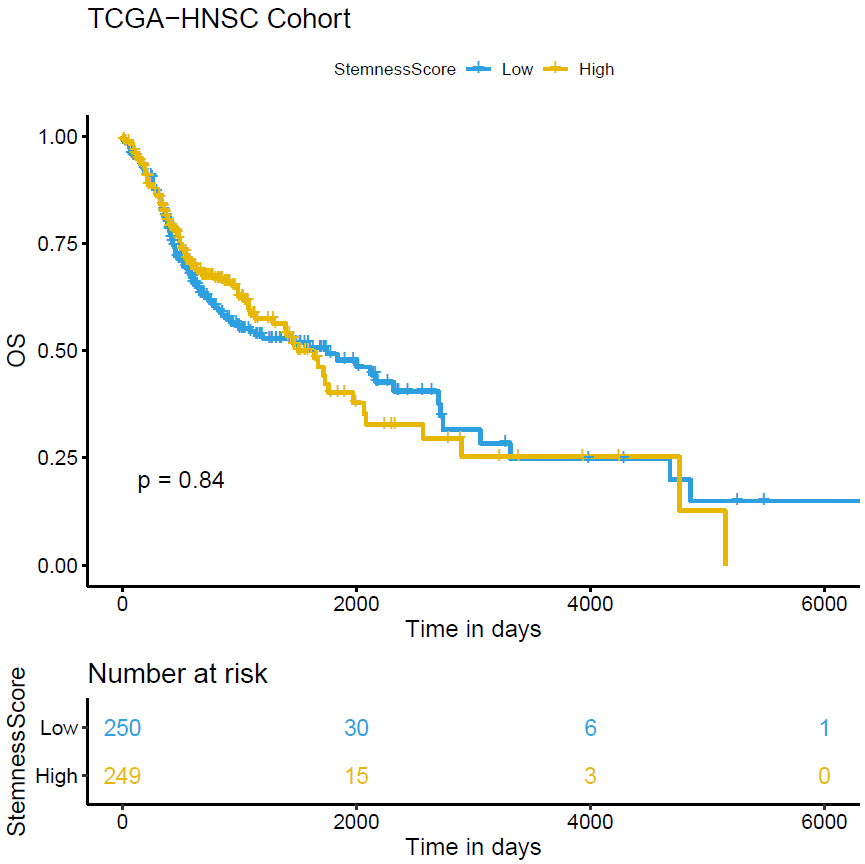


**Supplementary Figure 2.** Kaplan-Meier curves of stemness score in 499 TCGA-HNSCC cohort.


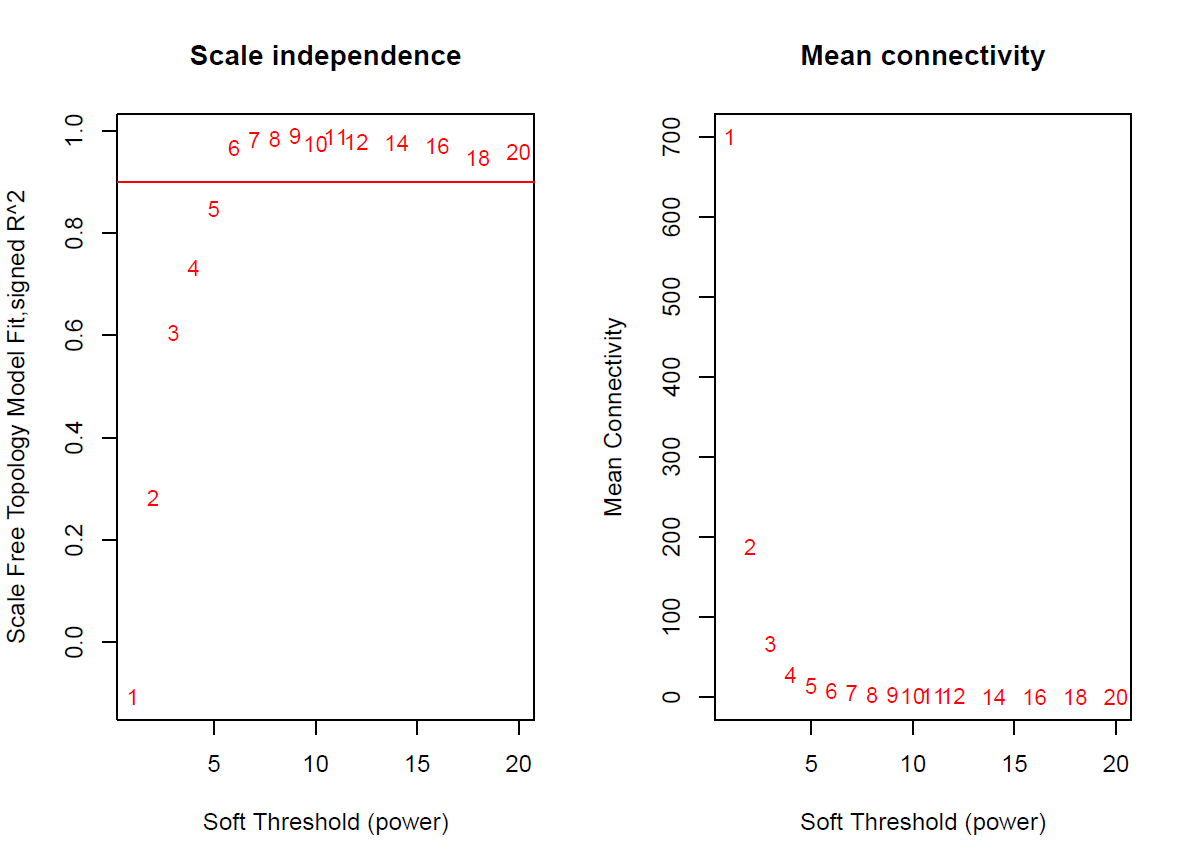


**Supplementary Figure 3.** Network topology for different soft-thresholding powers.


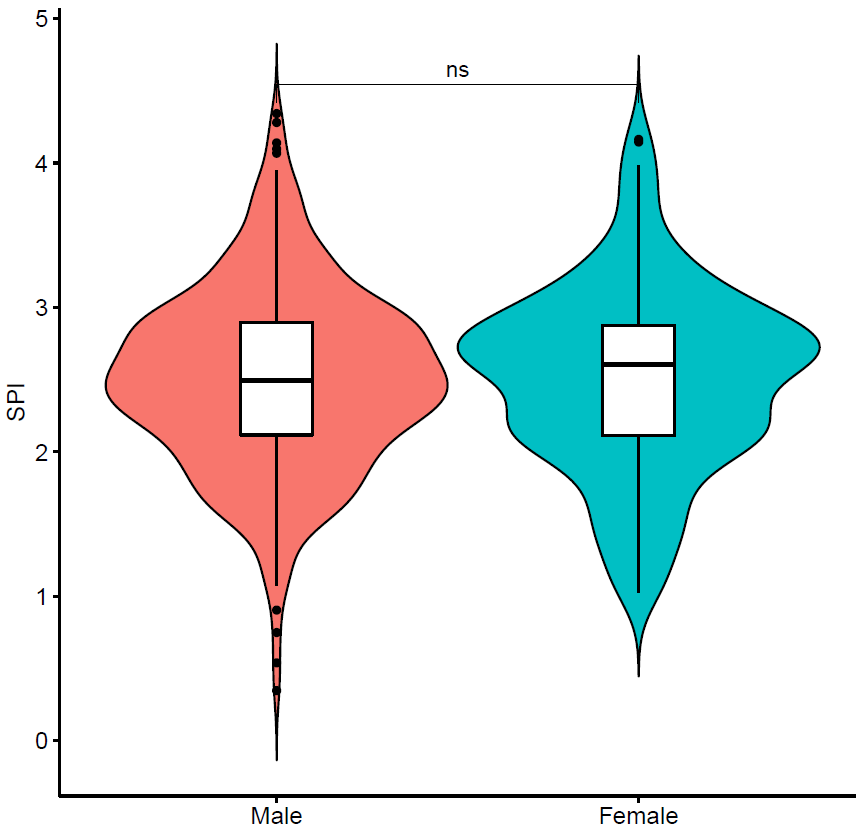


**Supplementary Figure 4.** No significant difference of stemness prognostic index (SPI) between male and female patients with HNSCC.


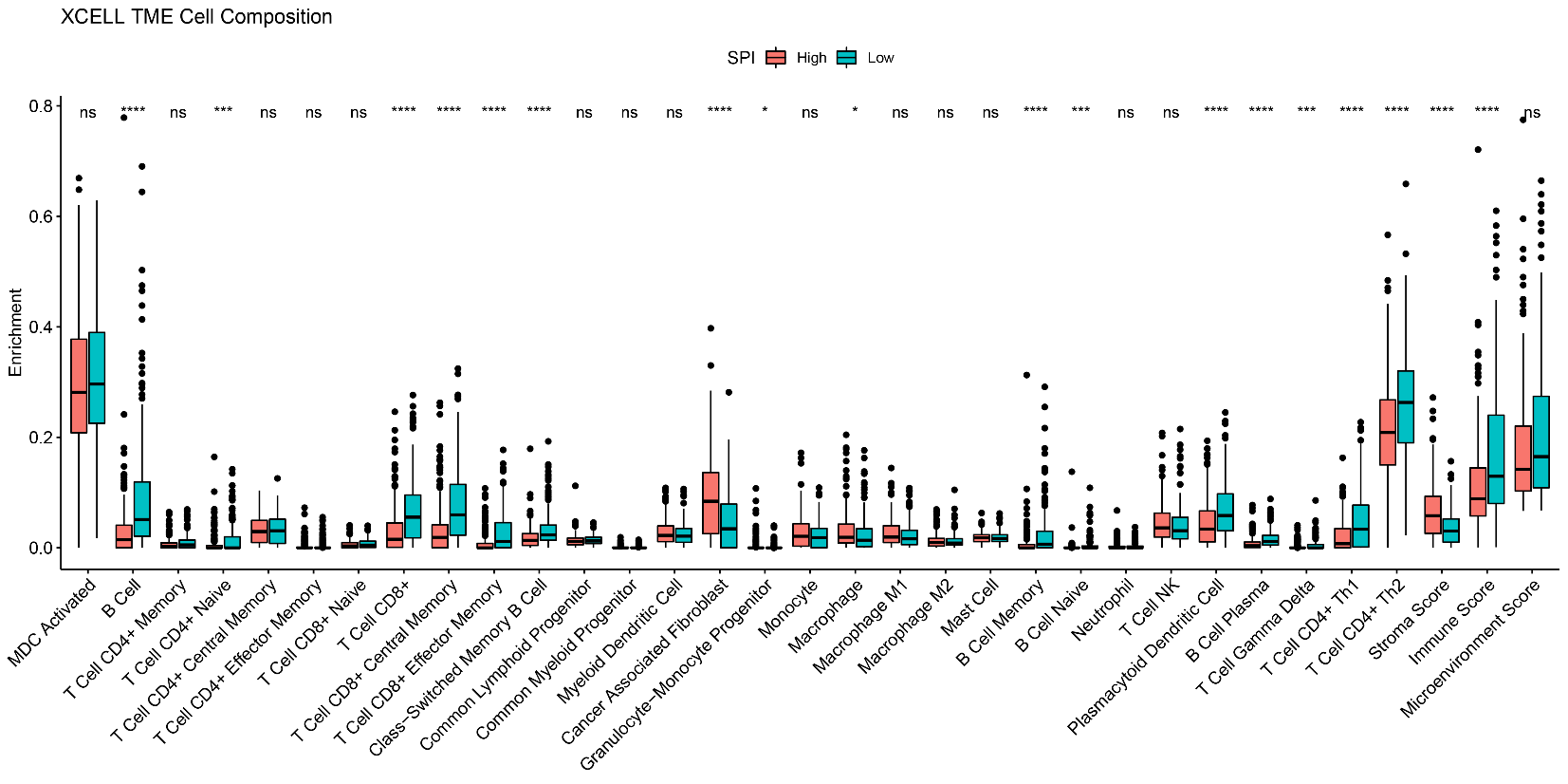


**Supplementary Figure 5.** Box plot showed the different enrichment level of non-tumor cells between different group based on XCELL algorithm.

## Supplementary Tables

**Supplementary Table 1.** Basic clinical characteristics of patients

| Factor | Level | TCGA | GSE65858 |
| --- | --- | --- | --- |
|  |  | Overall（N=499) | Overall（N=270) |
| Gender (%) | Female | 133(26.7) | 47(17.4) |
|  | Male | 366(73.3) | 223(82.6) |
| Age (Mean (SD^*^)) |  | 61.08(11.92) | 60.12(10.34) |
| Stage (%) | I | 25(5.8) | 18(6.7) |
|  | II | 69(16.0) | 37(13.7) |
|  | III | 78(18.1) | 37(13.7) |
|  | IVA | 247(57.3) | 155(57.4) |
|  | IVB | 11(2.6) | 16(5.9) |
|  | IVC | 1(0.2) | 7(2.6) |
| Prognosis (%) | Alive | 281(56.3) | 94(34.8) |
|  | Dead | 218(43.7) | 176(65.2) |

***SD:** Standard deviation

**Supplementary Table 2.** Multivariable analysis of five stemness-related genes signature in HNSCC

| Genes | Training TCGA set (n=332) | | | Validating TCGA set (n=167) | | | Total TCGA set (n=499) | | |
| --- | --- | --- | --- | --- | --- | --- | --- | --- | --- |
|  | HR | 95% CI | p value | HR | 95% CI | p value | HR | 95% CI | p value |
| SPOCK1 | 1.19 | 1.08-1.31 | **< 0.001** | 1.21 | 1.06-1.40 | **0.006** | 1.19 | 1.11-1.29 | **< 0.001** |
| BOC | 0.74 | 0.64-0.87 | **< 0.001** | 0.93 | 0.75-1.15 | 0.500 | 0.81 | 0.7149-0.91 | **< 0.001** |
| MME | 1.20 | 1.08-1.35 | **0.001** | 1.13 | 1.02-1.25 | **0.020** | 1.17 | 1.08-1.26 | **< 0.001** |
| GRIA3 | 0.86 | 0.77-0.96 | **0.007** | 0.78 | 0.67-0.90 | **0.001** | 0.83 | 0.77-0.91 | **< 0.001** |
| KNSTRN | 1.30 | 1.03-1.65 | **0.027** | 1.34 | 1.00-1.80 | **0.049** | 1.32 | 1.10-1.58 | **0.002** |

**Supplementary Table 3.** Multivariable analysis of training and validating sets of HNSCC

| Factor | TCGA training set (n=332) | | | TCGA validating set (n=167) | | | GSE65858 validating set (n=270) | | |
| --- | --- | --- | --- | --- | --- | --- | --- | --- | --- |
|  | HR | 95% CI | p value | HR | 95% CI | p value | HR | 95% CI | p value |
| Gender | 0.73 | 0.44-1.21 | 0.223 | 1.21 | 0.62-2.33 | 0.577 | 1.18 | 0.69-2.03 | 0.536 |
| Age | 1.02 | 1.00-1.05 | **0.023** | 1.04 | 1.01-1.07 | **0.015** | 1.03 | 1.01-1.05 | **0.010** |
| T stage | 1.44 | 0.90-2.31 | 0.128 | 1.41 | 0.80-2.49 | 0.230 | 2.78 | 1.68-4.57 | **< 0.001** |
| N stage | 1.07 | 0.65-1.74 | 0.799 | 0.95 | 0.54-1.69 | 0.873 | 1.12 | 0.69-1.81 | 0.645 |
| M stage | 2.17 | 0.63-7.43 | 0.217 | 4.36 | 0.45-42.62 | 0.206 | 2.47 | 1.05-5.82 | **0.039** |
| SPI^*^ | 2.63 | 1.74-3.98 | **< 0.001** | 2.14 | 1.43-3.20 | **< 0.001** | 1.85 | 1.16-2.95 | **0.010** |

*SPI: stemness prognostic index.
